# Characteristics of meconium impaction/retention in newborn foals: From 2006 to 2024

**DOI:** 10.1111/eve.14109

**Published:** 2025-01-29

**Authors:** S. Rivera Maza, R. C. Bishop, S. M. Austin, J. H. Foreman, P. A. Wilkins

**Affiliations:** Department of Veterinary Clinical Medicine, College of Veterinary Medicine, University of Illinois at Urbana-Champaign, Champaign, Illinois, USA

**Keywords:** horse, colic, enema, neonate, NICU

## Abstract

**Background:**

Meconium impaction/retention is a significant cause of colic in foals. Historically, limitations of both medical and surgical treatment are noted. Outcomes of meconium impaction/retention have not recently been reported.

**Objective:**

To describe case characteristics and outcomes in foals with meconium impaction/retention.

**Study design:**

Retrospective, single referral hospital.

**Methods:**

Medical records from 2006 to 2024 were searched for foals ≤3 days old with a history of straining to defecate, abdominal distention, colic, weakness and/or tail flagging. Signalment, presenting complaint, treatment, outcome and comorbidities were summarised and compared between groups based on sex and survival. Associations between comorbidities and survival were evaluated by zero-inflated Poisson regression.

**Results:**

Forty-three foals met the inclusion criteria. Male foals dominated (30/43; 70%). All foals were managed with enemas; phosphate (11/43; 30%), water with soap/lube (25/43; 58%), not specified 9/43 (21%), multiple types (8; 19%), acetylcysteine retention (1; 2%). Forty foals (93%) survived to discharge, and three (7%) were humanely euthanised. Of surviving foals, 37/40 (93%) responded fully to medical treatment. Surgical treatment was required in 4 foals (9%); 3/4 (75%) survived. Comorbidities were common including sepsis (10/43; 23%), pneumonia (10/43; 23%), failure of passive transfer (6/43; 14%) and hypoxic-ischaemic encephalopathy (5/43; 12%). Non-survivors (3/43; 7%) were euthanised due to sepsis, limb malformation and pneumonia.

**Main limitations:**

Small sample size and low number of non-survivors prevented meaningful statistical analysis.

**Conclusion:**

Medical management of meconium impaction/retention is successful in the great majority of cases. Prognosis depends on the comorbidities present.

## INTRODUCTION

Meconium impaction/retention is one of the most common causes of colic in newborn foals (Carr, 2014; MacKinnon et al., 2013). One of the earliest descriptions of successful treatment of meconium impaction called ‘birth plug stoppage’, appeared in a 1947 case report (Belonge, 1947). Prior to this report, meconium impaction/retention in Thoroughbred foals was considered to have high mortality, resulting primarily from rectal injuries associated with attempts to physically remove the ‘birth plug’. Treatment in the reported case included administration of three ounces of castor oil in mare’s milk and port wine, in addition to enemas of warm water and oil.

Meconium is composed of intestinal secretions, swallowed amniotic fluid and cellular debris. It is expected to be evacuated within the first 12–24 h following birth and ingestion of colostrum (Barr, 2007). Meconium impaction/retention is considered when the newborn foal attempts to defecate and fails, or passes only a small amount of meconium in the first 24 h of life (Pusterla et al., 2004). Dysmaturity, trauma at birth and other diseases have been associated with meconium impaction/retention (Hughes et al., 1996). In addition, meconium impaction/retention is reportedly more common in colts, which could be associated with anatomic differences: the pelvic inlet of colts is two-thirds the diameter of fillies, coupled with a convex pubis (Hughes et al., 1996; Ryan & Sanchez, 2005).

Clinical signs of meconium impaction/retention include abdominal distention, colic, weakness, straining to defecate, tail flagging, lack of faecal production and disinterest in suckling (MacKinnon et al., 2013). Digital examination and abdominal ultrasonography are commonly used techniques to confirm the presence of firm faecal material in the distal colon or rectum (Carr, 2014) and to aid in the identification of meconium in the large bowel. Impacted faecal material in the dorsal or transverse colon is considered a ‘high meconium impaction’ (Hughes et al., 1996). Diagnostic imaging can be used to support a diagnosis of meconium impaction: abdominal ultrasonography is most commonly used in current practice, while abdominal radiography and enemas with radiodense materials were more commonly used in the past (Rakestraw & Hardy, 2012).

Currently, medical management with enemas, such as warm water and soap, phosphate (Fleet Enema^®^; Fleet Laboratories), acetylcysteine, mineral oil or warm water and rectal lube, is performed in the great majority of cases. Adjunctive treatments can include administration of oral laxatives through a nasogastric tube (i.e. mineral oil), and/or oral medications such as lactase enzyme supplements (i.e. Lactaid^®^; McNeil Nutritionals LLC). Intravenous fluids with dextrose supplementation might be necessary for the provision of fluids and as an energy source as foals are generally kept from nursing. Analgesics may be provided for pain relief and to prevent self-trauma, and antibiotics are commonly instituted to treat or prevent concurrent infection (Magdesian, 2008). If foals show severe abdominal distension and pain that does not improve with medical management, transcutaneous trocarisation or surgical management may be indicated (Cable et al., 1997; Magdesian, 2008).

Historical reports cite poor survival following surgical treatment of meconium impaction, likely due to pre-existing or new sepsis (Adams et al., 1988; Crowhurst et al., 1975). Pusterla et al. (2004) reported good success using treatment with acetylcysteine retention enemas. However, there are no recent case studies evaluating and describing various treatments in a large population of foals with meconium impaction/retention. The objective of this study was to describe retrospectively the signalment, presentation, treatment, outcome and common comorbidities in newborn foals (0–3 days of age) with meconium impaction/retention at a single referral hospital.

## MATERIALS AND METHODS

### Sample collection

University of Illinois Veterinary Teaching Hospital electronic and paper medical records from 2006 to 2024 were searched for foals presented between 0 and 3 days of age. The criteria for inclusion were foals with a history of straining to defecate, abdominal distention, colic, weakness and/or tail flagging within 3 days of birth. Foals were excluded if there was no history of colic in the record or were older than 3 days of age at the time of presentation. Signalment, presenting complaint, physical examination findings, laboratory data, treatment(s), comorbidities and outcome were recorded. Historical data such as details of parturition, including dystocia, premature placental separation during delivery or placentitis were recorded if available in the medical record. Laboratory data recorded included: packed cell volume (PCV), total protein (TP), L-lactate, serum amyloid A (SAA), immunoglobulin G (IgG), blood glucose, blood gas, complete blood count (CBC), fibrinogen, blood chemistry and blood culture results (anaerobic and aerobic) when available.

Treatment was classified as medical or surgical management: medical management if meconium-associated colic was resolved with enemas or oral laxatives, surgical management if exploratory laparotomy was performed to treat meconium impaction-associated colic due to lack of resolution after medical therapies. Treatment details were recorded and included the number and type of enema(s), antibiotics (type and route of administration), IV fluids, nasogastric intubation (for gastric decompression and/or administration of enteral nutrition, fluids or mineral oil), and abdominal trocarisation or surgery if performed.

Enema type was categorised as: phosphate enema (Fleet Enema^®^; Fleet Laboratories), water with mild soap or lube enema or acetylcysteine enema. Standard practice for each type of enema is described briefly as follows. Commercial phosphate enemas (Fleet Laboratories) were administered by inserting the nozzle into the rectum gently and then rapidly squeezing the bottle to administer the phosphate-based solution. Warm water enemas were usually prepared as 150–250 mL warm water, depending on foal size, with either 50 or 100 mL of commercially available rectal lubricant or ~20 mL of a mild soap (such as liquid Ivory^™^; Procter & Gamble Co.) added. Warm water enemas were administered per rectum using soft tubing under gravity flow while the foals were in lateral recumbency. Acetylcysteine retention enemas were composed using a standard recipe. A buffered 4% acetylcysteine solution was made by adding 20 g of baking soda and 8 g of acetylcysteine to 200 mL of water. A 30F Foley catheter with a 30 mL balloon (Jorgensen Laboratories LLC) was placed into the rectum. The balloon on the catheter was enlarged, enema solution was administered by gravity flow and the tubing was clamped for ~20 min and then released.

Foals requiring trocarisation were sedated to effect, typically with 0.1 mg/kg bwt butorphanol IV, and restrained in lateral recumbency. The selected area within the right or left paralumbar fossa was based on the location of gas accumulation, as determined by ‘pinging’ or abdominal ultrasonography. The area was clipped and aseptically prepared, and 2–3 mL 2% lidocaine was injected subcutaneously at the intended puncture area. Puncture was performed using a sterile technique. In general, a 3.5-inch 18ga spinal needle (Becton, Dickinson & Co.) attached to a 32-inch IV extension set (Zoetis Inc.) was used for trocarisation. The free end of the extension set was placed into a container of water to monitor gas evacuation (via the production of bubbles). When gas decompression stopped, the spinal needle was withdrawn and digital pressure was applied to the puncture site.

If surgical treatment was performed, surgical diagnosis and specific lesion location were recorded. Non-meconium-associated disorders that compromised foals were identified in medical records and were determined as comorbidities. Diagnosis of coexisting diseases during hospitalisation was recorded: sepsis, pneumonia, flexural deformities, ruptured bladder (uroabdomen), orthopaedic infection, hypoxic-ischaemic encephalopathy (HIE) and failure of transfer of passive immunity (FPT). The outcome of each case was recorded as survival (survived to hospital discharge) or non-survival (euthanised or died). For neonates that were euthanised, the specific reason was recorded if available from the record.

### Statistical analysis

All analyses were performed in R version 4.1.2 (R Core Team, 2023; Jackman et al. 2009). Shapiro–Wilk test was performed to assess if data follows normal distribution (Gonzalez Estrada & Villasenor-Alva, 2009). Continuous data (rectal temperature, heart rate, respiration rate, age and laboratory data) were analysed using descriptive statistics; normally distributed data were summarised as mean ± standard deviation, nonparametric data by median (quartile 1, quartile 3). Each comorbidity was recorded as a binary variable (present or not present). Cases were also categorised as having a comorbidity diagnosis if they had one or more of the specific comorbidities. The relationship between sex and diagnosis of any comorbidity was assessed using the χ^2^ test (Wilkinson, 2011). The proportion of each comorbidity (sepsis, HIE, flexural deformities, ruptured bladder/uroabdomen, pneumonia and FPT) was calculated between survivors and non-survivors, and between colts and fillies.

## RESULTS

A total of 251 foals ≤3 days of age were identified and records were reviewed. A total of 43 foals (17%) met the inclusion criteria. Affected foals were 0.8 ± 0.9 days of age (approximately 18 h; range 0–3 days) at presentation, with colts (30; 70%) and fillies (13; 30%) aged 0.8 ± 0.9 and 0.7 ± 0.9 days (approximately19 and 16.5 h) old, respectively. Breed distribution of 43 included foals reflected hospital population: Standardbred (16; 37%), Thoroughbred (9; 21%), Quarter Horse (7; 16%), Belgian (2; 5%), Arabian (2; 5%), Tennessee Walker (2; 5%), Warmblood (1; 2%), Missouri Fox Trotter (1; 2%), Saddlebred (1; 2%), Miniature (1; 2%) and Dutch Harness (1; 2%). All foals were managed with at least one enema: phosphate enema (11/43; 30%), water with mild soap/lube enema (25/43; 58%), no enema type specified in 9/43 (21%), multiple enema types (8; 19%), retention enema with acetylcysteine (3; 7%). No enema-related complications were identified.

Surgical treatment was undertaken in seven foals (16%). Non-meconium-associated surgical problems were identified in three (7%) foals: umbilical haematoma and ruptured bladder repair (1); periosteal stripping for valgus deformity (1); umbilical resection (1). Four foals (9%) were surgically treated for meconium-associated colic. Records showed that six of 43 foals reported abnormal parturition: two (4.7%) cases of dystocia, three (7%) cases were premature at birth and one (2.3%) case was a prolonged gestation.

**FIGURE 1:**
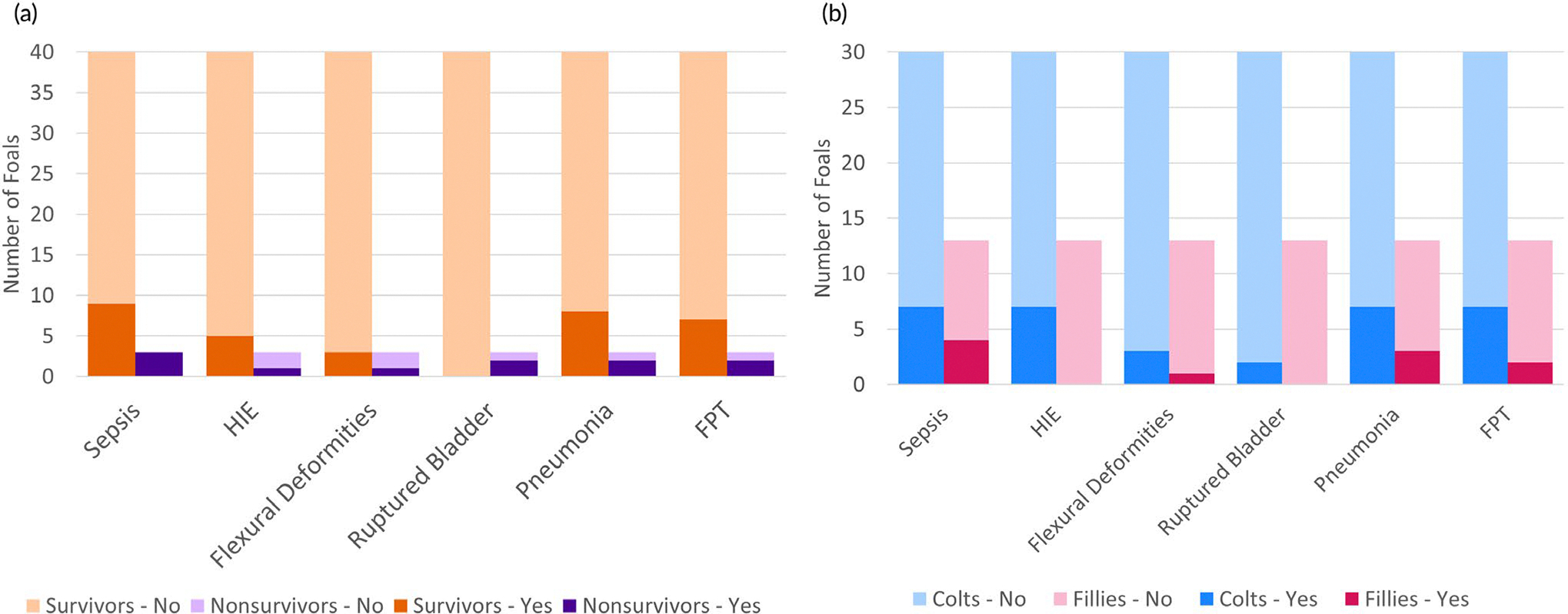
Stacked bar histogram of comorbidities in groups defined by (a) survival and (b) sex. Note that the total number of non-survivors was *n* = 3. There was no statistically significant difference between groups.

Forty foals (93%) survived hospital discharge, and three (7%) were euthanised due to poor prognosis and poor response to treatment associated with coexisting diseases/problems. Of surviving foals, 37 of 40 (93%) responded fully to medical treatment. Surgical treatment consisted of exploratory laparotomy via ventral midline approach, with manual decompression of meconium either via extraluminal lavage and digital manipulation per rectum or by manual decompression to the large colon and pelvic flexure enterotomy. Of foals treated surgically for meconium impaction, three of four (75%) survived. There were no post-operative complications recorded in surviving foals. The non-surviving foal was euthanised for recurrent signs of colic following surgical resolution of meconium impaction; a necropsy was not performed. Statistical analysis of variables associated with survival could not be performed due to the small number of non-surviving foals. Surviving neonates tended to have higher SAA, IgG, glucose, WBC and platelets in addition to lower PCV, heart rate, L-lactate and creatinine (Table 1), although there was a wide variation among survivors (Figure 1).

All non-surviving foals (three of three) had one or more comorbidities, whereas 19 of 40 (47.5%) surviving foals had comorbidities. There was no significant difference in occurrence of one or more comorbidity between sexes (*p* = 0.7): six of 13 fillies, and 16 of 30 colts had one or more comorbidity. The proportions of specific comorbidities within each group are summarised in Figure 1. There were no significant differences in the occurrence of any specific comorbidity between colts and fillies (Table 2).

## DISCUSSION

This retrospective study demonstrates that, overall, foals between 2006 and 2024 with a complaint of meconium impaction/retention had a high likelihood of survival as 40 of 43 foals (93%) survived hospital discharge. Early reports suggested poor outcomes with medical management, primarily due to attempts at physical removal using hands or tools or, later, the use of castor oil and alcohol oral treatments (van Wuijckhuise-Sjouke, 1984). More recent reports have demonstrated poorer survival rates associated with surgical management, primarily due to the development of post-operative complications such as sepsis and abdominal adhesions (Cable et al., 1997; Hughes et al., 1996; Vatistas et al., 1996).

Early attempts at medical treatment of meconium impaction/retention were associated with high complication rates. For example, complications were reported in 25 of 58 meconium impaction/retention cases, with 15 associated deaths in foals managed with castor oil and alcohol oral treatment in one report (van Wuijckhuise-Sjouke, 1984). Managing meconium impactions with castor oil is no longer recommended in newborn foals, or horses of any age, due to the increased risk of damage to epithelial cells, translocation of bacteria, sepsis and possible rectal perforation. Castor oil can produce excessive mucosal irritation and increase the risk of diarrhoea and colic (Magdesian, 2008). More recent reports demonstrate greatly improved survival of these cases with medical management, primarily repeated non-irritating enemas (Pusterla et al., 2004).

Surgical management has been used as a treatment for meconium impaction. Surgery should be considered and performed only if foals have failed to respond to aggressive medical therapy (Rakestraw & Hardy, 2012). According to Cable et al. (1997) foals receiving abdominal exploration appeared to suffer severe pain and lacked response to medical therapy. Post-surgical complications, such as sepsis and abdominal adhesions, are relatively common following abdominal exploration and relief of meconium impaction and have the potential to lead to euthanasia/death (Vatistas et al., 1996). In one study of 67 foals, two foals were surgically treated for meconium impaction, with one of the foals developing gastric ulceration and enteritis post-operatively that died before discharge. In another study of 24 foals, 16 were treated medically and eight foals underwent surgery due to poor response to medical treatment. Two of seven surgical cases were euthanised post-operatively due to repeated colic associated with serosal adhesions (Hughes et al., 1996). In addition, in Adams et al. (1988) 1 of 20 (5%) of foals required surgical management for meconium impaction; the author concluded that meconium impaction/retention should generally be managed medically with enemas, fluid and laxatives. During the 19 years included in this study, all 43 foals were treated with enemas and no enema-related complications were identified. Only four foals (9.3%) underwent surgical treatment of meconium impaction/retention following a poor response to medical management; 3/4 of those foals survived to discharge.

These data suggest that medical management of meconium impaction/retention is successful in the great majority of cases. Successful use of acetylcysteine retention enemas has been reported previously (Pusterla et al., 2004). Acetylcysteine cleaves the disulfide bonds in the mucoprotein molecules which helps with mucus breakdown and reduction of the viscosity of meconium (Rakestraw & Hardy, 2012). However, there are no recent case studies showing the success of commercially available enemas, such as phosphate enemas, or soap/lube and water enemas. In our study, warm water and soap/lube enemas, phosphate enemas and oral mineral oil, were used in the great majority of cases. Only 7% of foals received acetylcysteine retention enemas: one foal received only acetylcysteine with two foals receiving acetylcysteine enemas after soapy water enemas failed to resolve the impaction. Phosphate enemas, acetylcysteine solutions and a variety of lubes are commercially available, easy to use and inexpensive methods used to treat meconium impaction/retention (Burbidge, 2012).

In addition to a variety of enema types available for therapy, foals with meconium impaction/retention generally benefit from intravenous balanced crystalloid fluid administration and are prevented from nursing. An inability to control pain and the development of increased intrabdominal pressure frequently underlies the decision for exploratory laparotomy. Pain management can be challenging and, in cases where gas distention is present and contributing to pain and cardiovascular compromise, transabdominal trocarisation of the large bowel may be of benefit and allow additional time for resolution with medical management, avoiding some of the complications associated with exploratory laparotomy in equine newborn foals (Carr, 2014; Wong & Wilkins, 2024). Unfortunately, there was insufficient data regarding transcutaneous trocarisation in the case records to draw specific conclusions about outcomes in this study. Subjectively, in the authors’ experiences, trocarisation facilitates prolonged medical management, avoiding the potential complications of exploratory laparotomy.

In this study, colts outnumbered fillies 70% to 30%. It has been suggested that meconium impaction/retention is more prevalent in colts due to having a smaller size and shape of the pelvic inlet (Adams et al., 1988; Hughes et al., 1996). However, this finding may also reflect that newborn colts typically outnumber newborn fillies in presentation to a referral hospital for care (Adams et al., 1988). In this study, colts appeared to have more comorbidities including sepsis, failure of passive transfer and pneumonia, although the difference failed to reach statistical significance likely due to small numbers. While anatomical differences may contribute to the increased incidence of meconium impaction in colts, the overrepresentation of colts is also potentially attributable to increased birth rates (and subsequent presentation for NICU care) of colts compared to fillies.

Prognosis appeared to be dependent on the comorbidities present in this study, as the three non-surviving foals were euthanised due to deterioration/poor response of the comorbidity, rather than meconium impaction/retention. Comorbidities were more frequently present in non-surviving foals; however, this did not reach statistical significance due to the low number of non-survivors. The high survival rate overall invalidated our ability to perform meaningful statistical comparisons in some instances. Sepsis appeared to be the most common reason for non-survival, followed by failure of passive transfer, a risk factor for sepsis (Carr, 2014), pneumonia and other important infectious and inflammatory diseases (Carr, 2014; MacKinnon et al., 2013). Previous studies have reported that sepsis and failure of passive transfer were frequent concurrent diseases of neonates (Adams et al., 1988; Barr, 2007; Cable et al., 1997; MacKinnon et al., 2013; Pusterla et al., 2004).

## LIMITATIONS

Limitations of the current study include a relatively small sample size, very low number of non-survivors and lack of a comparison population of healthy foals without meconium impaction/retention or sick foals without presenting complaints of meconium impaction. There was an unequal sex distribution, as colts were overrepresented compared to fillies. Consistency and completeness of the medical records are a limitation of any retrospective study, with some information missing due to large portions of the medical records being handwritten until 2014. Some procedures, such as trocarisation, did not have an electronic charge code. The low number of non-survivors prevented meaningful comparisons between surviving and non-surviving foals but is reflective of the high success rate for medical management of meconium impaction. A larger sample size, prospectively collected from multiple sites would help us gain a better understanding of the characteristics of foals presenting with meconium impaction.

## CONCLUSIONS

Medical management of meconium impaction/retention is successful in most cases; transabdominal trocarisation of the large bowel may be useful for pain management and to obviate the need for surgical management. Surgical management should be performed only if foals do not respond to aggressive medical treatment. The prognosis appears to be largely dependent on comorbidities, but this needs to be examined in a larger cohort.

## Figures and Tables

**TABLE 1:** Summary statistics for foals categorised as survivors or non-survivors.

Parameter	Survivors [*n*]	Non-survivors [*n*]

PCV (%)	35.0 (32.0, 38.0) [32]	38.0 (31.0, 39.0) [3]
TP (g/dL)	5.07 ± 0.92 [32]	5.80 ± 1.15 [3]
Heart rate (beats/min)	117 ± 28.2 [38]	122 ± 40.1 [3]
L-lactate (mmol/L)	4.79 ± 6.68 [5]	14.0 [1]
SAA (μg/mL)	162 (62.7789) [8]	34.0 [1]
IgG (mg/dL)	800 (770,897) [10]	445 (267,622) [2]
Glucose (mg/dL)	113 ± 64.9 [21]	94.3 ± 7.09 [3]
Creatine (mg/dL)	1.30 (1.10, 2.00) [33]	1.35 (1.13, 1.58) [2]
WBC (×10^3^/μL)	6.90 ± 3.19 [34]	5.79 ± 1.39 [3]
Platelets (×10^3^/μL)	240 ± 7.41 [33]	159 ± 94.9 [3]

*Note*: Normally distributed data presented as mean ± SD, nonparametric as median (Q1, Q3). Number of individuals in [].

Abbreviations: IgG, immunoglobulin G; PCV, packed cell volume; SAA, serum amyloid A; TP, total protein; WBC, white blood cell count.

**TABLE 2:** Number and percentage of specific comorbidities between colts (*n* = 30) and fillies (*n* = 13).

Comorbidity	Colts	Fillies	*p*-value

Sepsis	23% (7)	31% (4)	0.70
Hypoxic-ischaemic encephalopathy	20% (6)	0% (0)	0.15
Pneumonia	23% (7)	23% (3)	1.00
Flexural deformities	10% (3)	8% (1)	1.00
Failure of passive transfer	23% (7)	15% (2)	0.99
Ruptured bladder	7% (2)	0% (0)	0.56

*Note*: *p*-Value reflects result of χ^2^ test; there was no statistically significant association between any individual comorbidity and sex.

## CLINICAL RELEVANCE

- The majority of meconium impaction/retention cases will resolve with medical management.
- Surgical relief of meconium impaction/retention is seldom required.
- The prognosis for meconium impaction/retention is primarily related to the prognosis for any coexisting comorbidity.
